# Comparative Study of Two Classification Criteria Sets in Real Clinical Practice for Behçet’s Disease

**DOI:** 10.3390/jcm14155559

**Published:** 2025-08-07

**Authors:** Rafael Gálvez-Sánchez, José Luis Martín-Varillas, Lara Sánchez-Bilbao, Iván Ferraz-Amaro, Elena Aurrecoechea, Diana Prieto-Peña, Ricardo Blanco

**Affiliations:** 1 Rheumatology, Hospital Marqués de Valdecilla, 39008 Santander, Spain; rafaelgalvez97@gmail.com (R.G.-S.);; 2 Immunopathology Group, Marqués de Valdecilla University Hospital-IDIVAL, 39011 Santander, Spain; 3 Rheumatology, Hospital de Laredo, 39770 Laredo, Spain; 4 Rheumatology, Hospital Universitario de Canarias, 38320 Tenerife, Spain; iferrazamaro@hotmail.com; 5 Rheumatology, Hospital de Sierrallana, 39300 Torrelavega, Spain

**Keywords:** Behçet’s disease, classification criteria, ISG, ICBD, sensitivity, clinical practice

## Abstract

**Background:** Behçet’s Disease (BD) was traditionally classified according to the International Study Group (ISG), where oral ulcers were mandatory. The International Team for the Revision of the International Criteria for BD (ICBD) introduced a scoring system instead. Our aim was to assess (a) sensitivity, (b) concordance between ISG and ICDB criteria in global and severe BD cases (ocular, vascular, and neurological), and (c) evaluate their clinical implications. **Methods:** Retrospective cohort study including 142 BD patients diagnosed in a well-defined population in Northern Spain, between January 1980 and November 2023. Both ISG and ICBD criteria were compared, sensitivity and concordance were assessed using Prevalence-Adjusted and Bias-Adjusted Kappa (PABAK) and the unadjusted Kappa. **Results:** A total of 142 BD patients diagnosed by expert rheumatologists (73 men; mean age of 36.4) were studied. Among them, 84 met ISG criteria, while 116 fulfilled ICBD criteria. Sensitivity of ISG and ICBD criteria in the overall cohort was (59.1% and 81.6%), respectively. Among patients with severe manifestations (ocular, vascular, or neurological), sensitivity increased to 71.2% for ISG and 92.5% for ICBD. Overall concordance was moderate (Kappa = 0.490), with 70.4% of patients classified identically. When adjusting prevalence and bias, concordance improved slightly (PABAK = 0.549). Of the 32 patients classified as BD exclusively by ICBD, 7 were receiving anti-TNF therapy, and 2 were receiving apremilast. **Conclusions:** The ICBD criteria demonstrated higher sensitivity than the traditional ISG criteria in classifying BD, particularly in severe cases. Classifying these additional patients under ICBD facilitated the initiation of on-label biologic treatments, potentially enhancing BD management, especially for severe cases.

## 1. Introduction

Behçet’s Disease (BD) is a systemic inflammatory disease characterized by blood vessels of varying sizes and clinically presenting with recurrent mucosal ulcers and uveitis [1]. BD has a distinctive geographical distribution, being more prevalent in countries around the Mediterranean and East Asia. Recent studies indicate that its prevalence and incidence are increasing in European and American countries, likely due to migratory flows and improved disease recognition [2,3,4,5,6,7].

Diagnosis of BD remains a challenge as no specific diagnostic tests are currently available. Furthermore, due to its clinical heterogeneity and prolonged asymptomatic periods, BD often has a diagnostic delay of several years [8].

For a better approach and classification of patients with BD, several criteria have emerged over the last decades. In 1990, the International Study Group for BD (ISG) [9] developed the most widely used set of classification criteria, in which the presence of oral ulcers is mandatory. These criteria had high specificity but low sensitivity.

Therefore, patients who did not experience at least three annual episodes of recurrent and painful oral ulcers but presented other characteristic clinical manifestations of BD could not be properly classified, and, in some cases, were not adequately treated.

As a consequence, an International Team for the Revision of the International Criteria for BD (ITR-ICBD) was formed two decades later [10]. This group published an updated set of criteria based on a scoring system, eliminating mandatory criteria and including additional clinical manifestations of BD that had not previously been considered. In this way, the sensitivity was improved while preserving the specificity of the previous criteria proposed by ISG.

The clinical manifestations of BD range from a mild entity with only mucocutaneous or joint involvement to a much more severe clinical condition with neurological, vascular, and ocular involvement, including blindness, stroke sequelae, aneurysms, and severe neurological deficits [11,12,13]. Therefore, the classification criteria should be especially useful in these serious cases.

Taking into account all the previous considerations, for overall BD and those with severe BD (ocular, vascular, and neurological), our aim was to assess (a) the sensitivity and (b) the concordance between ISG and ICBD criteria in both global and severe BD cases (ocular, vascular, and neurological involvement), and (c) to evaluate their clinical and therapeutic implications.

## 2. Materials and Methods

### 2.1. Study Design and Data Collection

We have performed a retrospective cohort study of 142 patients diagnosed with definitive or possible BD. The diagnosis of BD was established by expert rheumatologists specializing in this disease. They were diagnosed within a well-defined population in the region of Cantabria, Northern Spain, between January 1980 and November 2023. At the time of diagnosis, all the patients were residents of the municipalities belonging to Cantabria’s Health Area.

Patients were included consecutively. The only exclusion criterion was the presence of an alternative diagnosis that was considered more likely than BD, based on clinical records and expert judgment. The median follow-up time from diagnosis to last clinical assessment was 12.6 years (interquartile range [IQR]: 7.6–25.4 years). This prolonged observation period enabled the documentation of both early and late disease manifestations, including severe organ involvement.

We systematically reviewed medical records for demographic data (age at onset, sex, ethnicity) and detailed clinical manifestations of BD. Specifically, we recorded the history of oral aphthous ulcers, genital ulcers, skin lesions (including pseudofolliculitis, acneiform lesions, and erythema nodosum), ocular involvement (anterior uveitis, posterior uveitis, or panuveitis), vascular involvement (venous thromboses, arterial aneurysms, or superficial thrombophlebitis), neurologic involvement (central nervous system manifestations such as meningoencephalitis or brainstem syndrome, and peripheral neurologic involvement), and gastrointestinal involvement (e.g., ileocolonic ulcers) if present. We also noted any positive pathergy test results and HLA-B51 status, if available. For analytical purposes, we defined major organ involvement as the presence of ocular, neurologic, or major vascular manifestations at any point in the disease course, as these are often associated with more severe disease.

Patients were classified as BD according to either (a) ISG criteria (9): at least three episodes of painful oral ulcers in a year period, plus at least two of the following—recurrent genital ulceration, eye lesions (uveitis or retinal vasculitis), skin lesions (including erythema nodosum, pseudofolliculitis, or acneiform nodules in post-pubertal patients, or a positive pathergy test; or (b) ICBD criteria (10): the ICBD criteria were applied by assigning points for each manifestation—two points each for oral ulcers, genital ulcers, and ocular lesions, one point each for skin lesions, vascular involvement, and neurological involvement, and one point for a positive pathergy test.

To minimize misclassification and ensure reproducibility, ISG and ICBD criteria were applied algorithmically using structured formulas in Microsoft Excel, based on standardized data entry from the clinical records. These formulae were specifically adapted to reflect the requirements of each classification system: the point-based scoring model of the ICBD, and the ISG’s requirement of recurrent oral ulcers plus at least two additional typical features.

Patients who accumulated a total score of four or more points, according to the scoring system, were considered to fulfill the ICBD classification criteria. We considered a manifestation “present” if it had ever occurred during the disease course to maximize the likelihood of meeting criteria, except for pathergy, which was counted if a documented test was positive.

Data were gathered from the clinical charts according to a predefined protocol and then stored in a database. To minimize data entry errors, all data were systematically double-checked and reviewed for diagnosis confirmation.

### 2.2. Statistical Analysis

A comparative study was carried out to test the sensitivity and degree of agreement between the ISG and ICBD classification criteria. Results were expressed as numbers (percentages), mean ± standard deviation (SD), or median and interquartile range (IQR), as appropriate. Chi-squared and Fisher’s exact tests were used to compare categorical data. A *p*-value < 0.05 was considered statistically significant for all analyses. All statistical analyses were performed using IBM SPSS Statistics, version 26.0 (IBM Corp., Armonk, NY, USA).

The primary outcome was the sensitivity of each set of criteria—that is, the proportion of the 142 clinically diagnosed BD patients who were classified as BD by the criteria. We computed sensitivity for ISG and ICBD as (number of patients fulfilling the criteria/142) × 100%. Because our study did not include a control group of non-BD patients (given the rarity of BD, assembling a meaningful control cohort of similar conditions was beyond the study’s purpose), we did not directly measure specificity. Instead, our focus was on sensitivity and agreement between the two criteria. Sensitivity was calculated both for the general population with BD in our study and for individuals with severe BD involvement, defined as those with ocular, neurological, or vascular complications.

Inter-rater reliability beyond chance was assessed to evaluate the degree of agreement between ISG and ICBD classification criteria using Cohen’s Kappa coefficient. However, this measure can be compromised if Kappa is strongly affected by prevalence and bias between observers. To take these effects into account, PABAK (Prevalence- and Bias-Adjusted Kappa) adjustment was applied to the raw Kappa result, providing a more precise representation of the agreement. Kappa and PABAK values were interpreted using standard benchmarks (e.g., <0.20 poor, 0.21–0.40 fair, 0.41–0.60 moderate, etc.).

**Figure 1:**
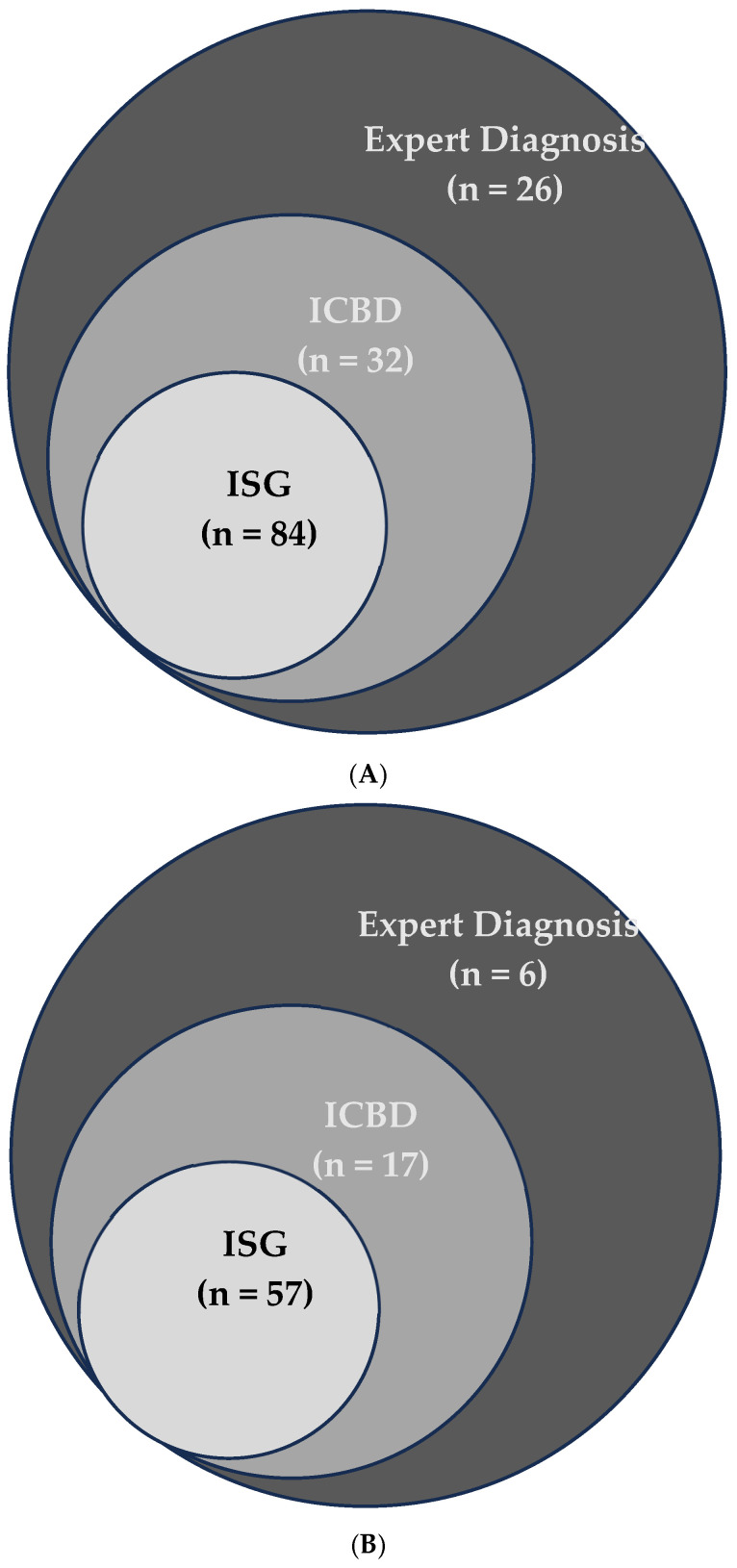
(**A**) Overlap of Behçet’s Disease cases detected by Expert Diagnosis, ICBD, and ISG criteria. The Venn diagram shows the unique and shared cases among the three methods. (**B**) Overlap of severe Behçet’s Disease cases detected by Expert Diagnosis, ICBD, and ISG criteria. The Venn diagram shows the unique and shared cases among the three methods.

To better understand the role of ocular, vascular, and neurological manifestations in the ISG and ICBD classification systems, and how they affect concordance between the two, we conducted a stepwise analysis. Each of these three clinical features was individually added to all patients—without altering their other symptoms—and then both ISG and ICBD criteria were applied to determine whether patients met one, both, or neither set of criteria. The agreement between the two systems was measured using the Kappa statistic. The same procedure was repeated by removing each manifestation separately from all patients and recalculating Kappa. This approach allowed us to evaluate the individual impact of each manifestation on the agreement between ISG and ICBD. To visually represent the overlap and unique classifications for each method under these scenarios, Venn diagrams (Figure 1A,B) were used.

### 2.3. Ethical Approval

This study was approved by the Cantabria Clinical Research Ethics Committee (2020.083) on 13 March 2020, and it was conducted in accordance with the Declaration of Helsinki. Given the retrospective nature of the study and the use of anonymous data, the ethics committee did not require informed consent.

## 3. Results

### 3.1. General Characteristics of the Cohort

We analyzed 142 patients (73 men and 69 women) diagnosed with BD by expert rheumatologists based on clinical judgment without applying formal classification criteria. The date of symptom onset was not consistently available in clinical records, and, therefore, the time elapsed until formal classification could not be calculated. The mean age at diagnosis was 36.4 ± 13.9 years. Baseline demographic characteristics, including age and sex, showed no significant differences between those fulfilling ISG and ICBD criteria (Table 1).

Regarding clinical features, oral aphthosis was the most frequent symptom (95.1%), followed by genital ulcers (62%), skin manifestations (64.1%), and joint involvement (61.3%). Among major organ involvement, ocular lesions were the most common (42.3%), followed by neurological (20.4%) and vascular involvement (13.4%).

When stratified by classification system, ocular involvement was observed in 55 patients (47.4%) classified by ICBD and in 49 patients (58.3%) classified by ISG. Neurological manifestations were present in 22 ICBD-classified patients (18.9%) and 13 ISG-classified patients (15.4%). Vascular involvement was seen in 17 patients (14.6%) under ICBD and 9 (10.7%) under ISG.

Among the 37 patients identified exclusively by ICBD, 17 (45.9%) had at least one severe organ manifestation. A statistically significant difference between ICBD and ISG groups was observed only in skin involvement frequency (88.1% in ISG vs. 68.1% in ICBD; *p* = 0.01), mainly due to a higher prevalence of erythema nodosum among ISG-classified patients.

### 3.2. Sensitivity in Overall and Severe BD

**Figure 2:**
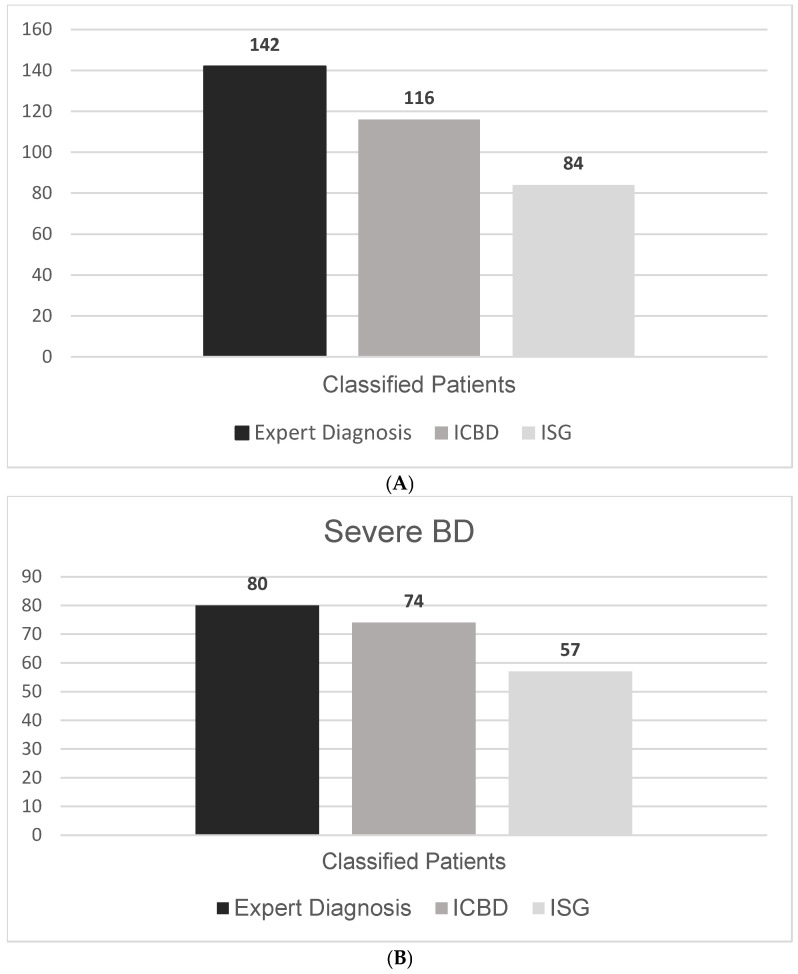
(**A**) Number of Behçet’s Disease cases identified by different classification methods. (**B**) Number of severe Behçet’s Disease cases identified by different classification methods.

Of the 142 patients evaluated, 84 (59.1%) fulfilled the ISG 1990 classification criteria. When the ICBD 2013 criteria were applied, the number increased to 116 (81.6%), reflecting a marked improvement in sensitivity. This difference represents a 22.5 percentage-point increase in sensitivity when using ICBD compared to ISG (Figure 2A). Notably, all patients fulfilling the ISG criteria also met the ICBD criteria, while 32 additional patients were classified only by ICBD.

A Venn diagram (Figure 1A) illustrates the distribution of patients fulfilling ISG, ICBD, or both sets of criteria. Among the 32 patients uniquely classified by the ICBD criteria, 17 (53.1%) had at least one severe organ involvement, defined as those with ocular, neurological, or vascular manifestations.

When we restricted the analysis to patients with severe Behçet’s Disease (n = 67), the ICBD criteria demonstrated an even greater advantage in sensitivity. ICBD classified 62 of these patients (92.5%), while ISG criteria identified only 48 (71.2%) (Figure 2B and Table 2). The overlap and differences between criteria among this subgroup are shown in Figure 1B.

### 3.3. Concordance Between ISG and ICBD for BD

The overall agreement between the ISG and ICBD classification criteria was classified as moderate, with a Cohen’s Kappa value of 0.490 (Table 2). Agreement was higher for positive cases (84.0%) compared to negative cases (61.9%). The global agreement between both systems was 77.5%. After adjusting for prevalence and bias, the Prevalence- and Bias-Adjusted Kappa (PABAK) increased to 0.549.

To further assess the influence of each major clinical feature on classification agreement, we systematically added or removed ocular, neurological, and vascular involvement from all patients and recalculated Kappa values (Table 3). The highest concordance occurred when ocular involvement was included in the dataset (PABAK = 0.788), whereas the lowest agreement was observed when vascular features were incorporated (PABAK = 0.338). Conversely, removing ocular involvement significantly lowered agreement (PABAK = 0.408).

### 3.4. Clinical Impact: Relationship with Biological Therapy

A total of 36 patients (25.3%) received anti-TNF therapy during the course of their disease, most commonly adalimumab (20 patients, 14.1%) and infliximab (15 patients, 10.5%) (Table 1). Among them, 28 (77.8%) fulfilled ISG criteria and 35 (97.2%) fulfilled ICBD criteria. Only one patient treated with anti-TNF agents did not meet either classification criterion.

Notably, 8 of the 36 patients (22.2%) who received anti-TNF therapy did not meet ISG criteria at the time of treatment initiation, while only 1 (2.8%) did not fulfill ICBD criteria. Among the 37 patients classified exclusively by ICBD and not by ISG, 7 received anti-TNF therapy (4 with adalimumab, 3 with infliximab), and 2 were additionally treated with apremilast. Five of these seven ICBD-only patients had ocular involvement.

## 4. Discussion

Our study highlights the ongoing challenge of accurately and efficiently classifying Behçet’s Disease (BD), particularly in patients with severe organ involvement.

We focused on comparing the two most commonly used classification criteria for BD in routine clinical practice. As demonstrated by ITR-ICBD et al. [10] and later reaffirmed by Zhenyu Zhong et al. [14], our study supports the higher sensitivity of the ICBD criteria versus the initial ISG criteria for BD diagnosis [10,14]. However, differences in prevalence may be associated with clinical variability in the spectrum, leading to changes in sensitivity [15,16]. Therefore, it was necessary to assess the sensitivity of these classification criteria in our setting (Spain), because regional differences in the presentation and prevalence of BD can influence the effectiveness of classification tools.

Cohen’s Kappa index indicates the degree of agreement between two independent observers on nominal variables. Its value ranges from −1 (perfect disagreement), 0 (null agreement), to 1 (perfect agreement). The following Kappa values are commonly used, as proposed by Landis and Koch (9), to classify the following degrees of agreement: no agreement (<0), insignificant (0 to 0.2), low (0.2 to 0.4), moderate (0.4 to 0.6), good (0.6 to 0.8), and very good (0.8 to 1) [17]. The agreement between ISG and ICBD criteria was classified as moderate, with a Kappa of 0.490 (95% CI: 0.356–0.623) and PABAK of 0.549.

Zhenyu Zhong et al. [14] demonstrated in a case–cohort and nested case–control study with a total of 2440 and 2224 participants that the highest diagnostic consistency was observed between ISG criteria and ICBD criteria for cases with scores ≥ 5 on the ICBD scale (Kappa = 0.999; *p* < 0.001), improving sensitivity and specificity, and suggesting that optimizing the diagnostic threshold would be a way to improve ICBD performance.

Therefore, by increasing the requirements to meet diagnostic criteria, the specificity of the tools is likely to improve. In clinical trials, specificity is often considered more critical, as every patient enrolled must truly have BD. However, for early or mild disease in daily clinical practice, classification criteria play a less significant role in treatment decisions, since rheumatologists ultimately rely on their clinical judgment, imaging, and other investigations. In our cohort, we applied the official ICBD threshold of ≥4 points, as per current recommendations. Although we did not formally test higher cutoffs, increasing the threshold would likely reduce sensitivity, especially in patients with isolated severe manifestations but few mucocutaneous symptoms. Therefore, any upward adjustment may risk underclassification in such cases.

Investigating other diagnostic alternatives that can help an expert’s clinical judgment in the diagnostic process is necessary, as diagnosing BD poses a significant challenge in daily practice, especially in patients presenting with only major organ involvement, with or without oral ulcers. Alibaz-Oner et al. [18] demonstrated that there are other indicative data, such as CFV thickness measurement, that can be helpful, with sensitivity and specificity exceeding 80% for a cut-off value of 0.5 mm when compared against other disease groups and ethnic populations. Mina Kiafar et al. [19] performed a retrospective analysis in which patients in the BD group had a significantly higher prevalence of a family history of BD (9/40 vs. 5/93; *p* < 0.001), and Lourdes Ortiz-Fernández et al. [20] estimated a BD heritability to be at least 16%. Taken together, these findings suggest that markers such as CFV thickness and positive family history could be integrated as supportive elements in future classification systems, particularly to improve sensitivity in atypical or oligosymptomatic cases.

Unfortunately, the high degree of heterogeneity of BD limits the use of standardized diagnostic criteria, and its diagnosis often requires additional adaptations depending on the geographic area, the prevalence of the disease, the medical specialty, the characteristics of local practice, and the need for new tools. This is why the clinical judgment of an expert rheumatologist is crucial in diagnosing BD. Although classification criteria facilitate epidemiological and research studies, therapy initiation in real-world practice is not strictly dependent on them. Since early diagnosis of BD is crucial, any delay in treatment may lead to severe manifestations. Few studies have addressed the mortality rate of BD. Among 2031 patients from Japan, 31.7% experienced clinical deterioration and 0.9% died during a one-year follow-up. In Turkey, nearly 10% of deaths among 428 patients were due to major vessel disease and neurologic involvement [21]. We did not collect mortality data in our cohort, which limits comparisons with previously reported rates.

In this context, our study provides additional insight into the practical application of classification criteria in real-world settings. One of its main strengths is the analysis of a well-defined cohort over a long observation period, allowing a comprehensive assessment of disease presentation and evolution. The inclusion of patients diagnosed by expert rheumatologists adds further robustness to the classification accuracy. However, certain limitations should be acknowledged. The retrospective design may have introduced information bias, and the absence of a control group precludes specificity analysis. This limitation is particularly relevant when considering the potential decrease in specificity associated with the ICBD criteria. In clinical practice, this may lead to overclassification or unnecessary immunosuppressive treatment in borderline cases, highlighting the need to interpret these criteria alongside expert clinical judgment. Additionally, applying the classification criteria across the entire disease course, rather than at disease onset, may have overestimated the sensitivity of both ISG and ICBD systems. Another limitation is the lack of reliable data regarding the timing of symptom onset, which prevented us from evaluating the delay between disease onset and fulfillment of classification criteria. These factors may have led to an overestimation of sensitivity and should be considered when interpreting the results. Moreover, since this study was conducted in a single region of Northern Spain with relatively low BD prevalence compared to endemic areas, the external validity of the findings may be limited. Moreover, even within a single country, regional and ethnic variability may influence BD presentation, and our results should be validated in other populations before generalizing.

## 5. Conclusions

ICBD criteria showed greater sensitivity than ISG, especially due to the absence of oral ulcers in certain patients. Classifying these additional patients under ICBD facilitated the initiation of approved biologic treatments (e.g., monoclonal anti-TNF), potentially improving the management of BD, particularly in patients with ocular involvement and without oral ulcers.

These results highlight the practical advantages of using more inclusive classification criteria in routine clinical care. By identifying patients who would otherwise be missed by ISG, ICBD may help avoid treatment delays and reduce the risk of severe complications. Future multicenter and prospective studies including cohorts from different geographical regions will be essential to validate these findings and assess the applicability of both criteria in diverse clinical and ethnic contexts.

## Figures and Tables

**Table 1:** Main clinical features according to different classification criteria, with emphasis on ocular involvement and biological treatments.

	Expert Diagnosis (N = 142)	ISG Criteria (N = 84)	ICBD Criteria (N = 116)	ISG vs. ICBD *p* Value
**Demographic data**				
Age, mean (SD)	36.4 (13.9)	35 (12.8)	37 (13.2)	
Gender, n (%)				
Male	73 (51.4)	41 (48.8)	57 (49.1)	0.80
Female	69 (48.6)	43 (51.2)	59 (50.9)	0.59
**Clinical manifestations n (%)**				
Oral aphthosis	135 (95.1)	84 (100)	115 (99.1)	0.99
Recurrent (≥3 times/year)	115 (81)	72 (85.7)	102 (87.9)	0.80
Genital aphthosis	88 (62)	67 (79.7)	88 (75.8)	0.63
Skin manifestations	91 (64.1)	74 (88.1)	79 (68.1)	0.01
Ocular involvement	60 (42.3)	49 (58.3)	55 (47.4)	0.13
Uveitis	52 (36.6)	42 (50)	47 (40.5)	0.18
Anterior	20 (14.1)	20 (23.8)	20 (17.2)	0.25
Intermediate	3 (2.1)	0	2 (1.7)	0.51
Posterior	14 (9.9)	11 (13.1)	13 (11.2)	0.68
Panuveitis	14 (9.9)	10 (11.9)	11 (9.5)	0.58
Unilateral	29 (20.4)	25 (29.8)	28 (24.1)	0.37
Bilateral	23 (16.2)	17 (20.2)	19 (16.4)	0.49
Dry eye	8 (5.6)	7 (8.3)	8 (6.9)	0.71
Scleritis	0	0	0	0.99
Episcleritis	4 (2.8)	3 (3.6)	4 (3.4)	0.99
PUK	1 (0.7)	1 (1.2)	1 (0.86)	0.90
Joint manifestations	87 (61.3)	52 (61.9)	71 (61.2)	0.99
Neurological manifestations	29 (20.4)	13 (15.4)	22 (18.9)	0.65
Vascular manifestations	19 (13.4)	9 (10.7)	17 (14.6)	0.54
**Complementary tests n (%)**				
Pathergy positive test	7 (5)	6 (7.1)	6 (5.1)	0.78
HLA 51 positive	51 (36)	25 (29.7)	38 (32.7)	0.76
**Treatment, n (%)**				
Anti-TNF treatment	36 (25.3)	28 (33.3)	35 (30.1)	0.63
Infliximab	15 (10.5)	12 (14.2)	15 (12.9)	0.78
Adalimumab	20 (14.1)	15 (17.8)	19 (16.3)	0.78
Etanercept	1 (0.7)	1 (1.2)	1 (0.8)	0.99
Apremilast	10 (7)	7 (8.3)	9 (7.7)	0.88
Anti-IL6r (Tozilizumab)	1 (0.7)	0	0	0.99

**Table 2:** Comparison of ISG and ICBD classification criteria performance in classifying Behçet’s Disease.

	ISG	ICBD
Sensitivity (%)	59.1	81.6
Sensitivity in severe cases (%)	71.2	92.5
Positive specific agreement (%)	84.0 (78.3–88.4)	
Negative specific agreement (%)	61.9 (51.2–71.6)	
Overall agreement (%)	77.5 (69.9–83.6)	
Kappa (95% CI)	0.490 (0.356–0.623)	
PABAK	0.549	

**Table 3:** Variation in concordance between ISG and ICBD classification criteria when each severe manifestation is systematically added to or removed from all 142 patients.

Condition	Kappa (Added)	PABAK (Added)	Kappa (Removed)	PABAK (Removed)
Ocular	0.405 (0.176–0.634)	0.788	0.443 (0.326–0.561)	0.408
Vascular	0.216 (0.103–0.330)	0.338	0.559 (0.427–0.690)	0.605
Neurological	0.235 (0.118–0.353)	0.352	0.507 (0.374–0.640)	0.563

## Data Availability

All data are contained within the article. Additional data are available from the corresponding author upon reasonable request.

## Abbreviations

BD: Behçet’s Disease; ISG, International Study Group; ICBD, International Criteria for Behçet’s Disease; TNF, tumor necrosis factor.
